# Shifting the Circadian Rhythm of Feeding in Mice Induces Gastrointestinal, Metabolic and Immune Alterations Which Are Influenced by Ghrelin and the Core Clock Gene Bmal1

**DOI:** 10.1371/journal.pone.0110176

**Published:** 2014-10-16

**Authors:** Jorien Laermans, Charlotte Broers, Kelly Beckers, Laurien Vancleef, Sandra Steensels, Theo Thijs, Jan Tack, Inge Depoortere

**Affiliations:** Gut Peptide Research Lab, Translational Research Center for Gastrointestinal Disorders, KU Leuven - University of Leuven, Leuven, Belgium; Simon Fraser University, Canada

## Abstract

**Background:**

In our 24-hour society, an increasing number of people are required to be awake and active at night. As a result, the circadian rhythm of feeding is seriously compromised. To mimic this, we subjected mice to restricted feeding (RF), a paradigm in which food availability is limited to short and unusual times of day. RF induces a food-anticipatory increase in the levels of the hunger hormone ghrelin. We aimed to investigate whether ghrelin triggers the changes in body weight and gastric emptying that occur during RF. Moreover, the effect of genetic deletion of the core clock gene Bmal1 on these physiological adaptations was studied.

**Methods:**

Wild-type, ghrelin receptor knockout and Bmal1 knockout mice were fed *ad libitum* or put on RF with a normal or high-fat diet (HFD). Plasma ghrelin levels were measured by radioimmunoassay. Gastric contractility was studied *in vitro* in muscle strips and *in vivo* (^13^C breath test). Cytokine mRNA expression was quantified and infiltration of immune cells was assessed histologically.

**Results:**

The food-anticipatory increase in plasma ghrelin levels induced by RF with normal chow was abolished in HFD-fed mice. During RF, body weight restoration was facilitated by ghrelin and Bmal1. RF altered cytokine mRNA expression levels and triggered contractility changes resulting in an accelerated gastric emptying, independent from ghrelin signaling. During RF with a HFD, Bmal1 enhanced neutrophil recruitment to the stomach, increased gastric IL-1α expression and promoted gastric contractility changes.

**Conclusions:**

This is the first study demonstrating that ghrelin and Bmal1 regulate the extent of body weight restoration during RF, whereas Bmal1 controls the type of inflammatory infiltrate and contractility changes in the stomach. Disrupting the circadian rhythm of feeding induces a variety of diet-dependent metabolic, immune and gastrointestinal alterations, which may explain the higher prevalence of obesity and immune-related gastrointestinal disorders among shift workers.

## Introduction

Not only the amount or type of food we ingest, but also the timing of food consumption seems to play a crucial role in the development of obesity and associated metabolic disorders [1]. In our 24-hour society, people are voluntarily shifting their normal activity patterns to unusual times of day, for instance by shift working or frequent time zone traveling. As a result, they are seriously compromising their circadian system, an evolutionary conserved timekeeping mechanism that generates daily behavioral rhythms that allow organisms to actively anticipate and adapt to predictable environmental changes, thereby increasing the likelihood of survival [2]. In mammals, this timing system is comprised, in a hierarchical way, of a hypothalamic master clock and peripheral oscillators in numerous body cells. At the molecular level, circadian rhythms depend on the concerted co-expression of a set of clock genes (Clock, Bmal1, Per1-3, Cry1-2) [2]. These genes participate in transcription-translation feedback loops, through which they not only regulate their own activity, but also that of downstream clock-controlled genes.

In order to maintain a near 24 h-rhythm, circadian clocks need to be entrained to daily environmental cues. Under normal circumstances, the master clock is synchronized by the light/dark-cycle and will further entrain the peripheral clocks. However, during restricted feeding (RF), when food availability is limited to short and abnormal times of the day, the phase of peripheral clock gene expression becomes uncoupled from the master clock and shifts in order to realign with mealtime [3]. The entrainment of these peripheral clocks by feeding cues is still present in mice given lesions in the master clock [4], suggesting that the circadian function of feeding depends on separate food-entrainable oscillators (FEOs). The FEOs predict food availability and ensure that the organism retains a positive energy balance, even if food is available at unusual times. The anatomical location of these FEOs remains elusive despite extensive research. Recently, LeSauter *et al.* found that the expression of ghrelin and of the clock genes PER1 and PER2 within the ghrelin-secreting cells of the stomach was rhythmic and entrained by food availability [5]. Hence, they proposed that these ghrelin-secreting cells might serve as one of the FEOs, producing a timed ghrelin output signal that acts widely at both brain and peripheral sites.

Ghrelin is a 28-amino acid peptide mainly secreted by the stomach [6]. The octanoylation of ghrelin at Ser^3^ by ghrelin O-acyltransferase (GOAT) is crucial to its biological activity. Besides being the only circulating hormone that stimulates food intake, ghrelin increases body weight by preventing fat utilization, accelerates gastric emptying and initiates hunger contractions in the fasted state [7]. Plasma ghrelin levels fluctuate diurnally, with a nocturnal elevation in humans [8]. In addition, levels surge during fasting and decrease postprandially to dictate the timing of meals [9], [10]. Studies in animals subjected to RF have highlighted this tight relationship between ghrelin and timing of feeding. Indeed, a preprandial rise in plasma ghrelin levels is observed in anticipation of a scheduled meal in rodents [11], [12]. Nevertheless, although controversial, the majority of the studies in ghrelin receptor knockout (GHSR-KO) and ghrelin knockout mice reveal that ghrelin and its receptor are not necessary for food-anticipatory behavior [13].

This study aimed to examine whether ghrelin might serve as an output signal of the FEO that triggers physiological adaptations to alterations in the circadian rhythm of feeding induced by RF. Here, we showed for the first time that RF not only results in changes in food intake, body weight and gastric emptying, but also induces alterations in gastric proinflammatory cytokine expression and myeloperoxidase (MPO) activity. Studies in wild-type (WT) and GHSR-KO mice revealed that ghrelin was merely involved in the restoration of body weight loss during RF. Next, we investigated whether the core clock gene Bmal1 might serve as the key driver behind the adaptations by studying the effects of RF in Bmal1 knockout (Bmal1-KO) mice, which is the only single clock gene knockout in which the mouse loses all rhythmic behavioral activity [14]. We found that Bmal1 not only determines the extent of body weight restoration during RF, but also defines the type of infiltrating immune cells and influences the changes in contractility prior to food availability during RF with a high-fat diet.

## Materials and Methods

### Ethics Statement

All mouse experiments were approved by the Ethical committee for Animal Experiments of the KU Leuven (project number P106/2011). All efforts were made to minimize animal suffering. After being anesthetized using a mixture of ketamine and xylazine, mice were sacrificed by decapitation.

### Animals

Breeding couples of mice heterozygous for GHSR and Bmal1 were kindly provided by Janssen Pharmaceutica (Beerse, Belgium) [15] and R. Lijnen (KU Leuven, Leuven, Belgium) [16], respectively. All KO mice and their WT littermates were bred in the animal facility of the KU Leuven and genotyped by polymerase chain reaction (PCR) analysis performed on total genomic DNA from the tail. Mice were housed (20–22°C) under a 12-h/12-h light/dark-cycle (lights on at 7 AM) and had *ad libitum* access to food and drinking water, unless stated otherwise.

### Diets

During RF experiments, mice were fed normal chow (Ssniff R/M-H, Ssniff Spezialdiäten GmbH, Soest, Germany; 9% kcal from fat) or canola oil-enriched chow (COEC) (powdered normal chow mixed with canola oil, 4∶1 wt/wt; 47% kcal from fat). When using COEC, mice were allowed to habituate to the newly introduced diet before starting the actual experiment.

### Experimental design

#### Standard RF schedule

**Figure 1:**
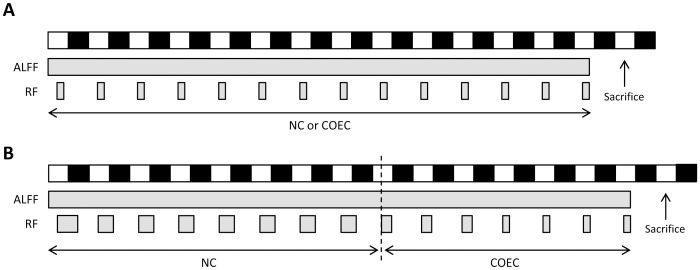
Restricted feeding protocols. (**A**) Scheme of the standard RF schedule in which food availability was limited to 4 hours a day (ZT 5–9) from day 1 until day 14. White and black bars represent times of lights on and off, respectively. Gray bars represent times of food availability of either normal chow (NC) or canola oil-enriched chow (COEC). (**B**) Scheme of the adapted RF schedule in which food availability was gradually decreased during the experiment. Both groups received NC for 8 days (RF group: 1 day 12 h food available (ZT 5–17) and 7 days 9 h food available (ZT 5–14)) and COEC during the remaining 7 days (RF group: 3 days 6 h food available (ZT 5–11) and 4 days 4 h food available (ZT 5–9)). Note that the time of onset of food availability is the same each day (ZT 5).

Mice were randomly assigned to either of two groups, subjected to different feeding regimes during two weeks. The RF group only had access to food for 4 hours between *zeitgeber* time (ZT) 5 and 9 (where ZT 0 is lights-on by convention), while the other group received food *ad libitum* (Figure 1*A*). In parallel with the RF group, food was removed from the *ad libitum* group at ZT 9 the day before being sacrificed. Hence, this group was termed *ad libitum* fed fasted (ALFF). A third group of mice, hereinafter referred to as the RF+ALFF group, was first subjected to two weeks of RF and then refed *ad libitum* during a period of four weeks. All mice were sacrificed at ZT 4.5.

#### Adapted RF schedule with gradually decreased food availability

WT and Bmal1-KO mice were either fed *ad libitum* or subjected to a RF schedule in which food availability was gradually decreased and chow was enriched with canola oil to increase the caloric content of the meal [17] (Figure 1*B*). Because of a low tolerance rate of the *ad libitum*-fed Bmal1-KO mice towards the amount of fat in the COEC, resulting in 80% mortality, both ALFF and RF mice received normal chow during the first 8 days and COEC during the next 7 days. All mice were sacrificed at ZT 4.5 after a 19.5 h fast.

### Ghrelin radioimmunoassay

Blood samples (1 mg/mL EDTA and 4 mM AEBSF) were centrifuged and acidified (10% HCl). After extraction on Sep-Pak C18 column, radioimmunoassay was performed as previously described [18].

#### Gastric emptying

Gastric emptying was measured using the ^13^C octanoic acid breath test, as described previously [15], [19]. T_half_ is the time point at which 50% of the total amount of ^13^CO_2_ was exhaled.

### *In vitro* contractility studies

Mucosal-free fundic smooth muscle strips were suspended along their circular axis in a Krebs-filled tissue bath. Concentration-response curves to acetylcholine (ACh) (10^−9^ to 10^−4^ mol/L) were established isometrically. The maximal tension (g/mm^2^) and concentration necessary to induce 50% of the maximal contraction (EC-50) were calculated. Neural responses were elicited by electrical field stimulation (EFS) (pulse duration 1 ms, train duration 10 s, amplitude 6 V) at increasing frequencies (0.5–16 Hz) [20]. On-relaxations were expressed relative to the response induced by 10^−5^ mol/L nitroglycerin. The area under the curve (AUC) of the off-contractions was expressed as g/mm^2^.

### Quantitative real-time PCR

Total RNA was isolated from stomach using the Qiagen RNeasy Mini Kit (Qiagen, Hilden, Germany) and reverse transcribed to cDNA using SuperScript II Reverse Transcriptase (Invitrogen, Carlsbad, CA, USA). Quantitative real-time PCR was performed as previously described [15]. Primer sequences were as follows: GAPDH: forward CCCCAATgTgTCCgTCgTg, reverse gCCTgCTTCACCACCTTCT; IL-1β: forward gACCTTCCAggATGAggACA, reverse TCCATTgAggTggAgAgCTT; IL-6: forward CCATAgCTACCTggAgTACATg, reverse TggAAATTggggTAggAAggAC; IL-1α: forward gAgAgCCgggTgACAgTATC, reverse ACTTCTgCCTgACgAgCTTC; ghrelin: forward CCAgAggACAgAggACAAgC, reverse ACATCgAAgggAgCATTgAA; GOAT: forward ACCCgggCCAggTACCT, reverse ACCCATggCAgCAAAAgC.

### MPO activity assay

Fundic MPO activity was determined using the O-dianisidine-H_2_O_2_ method. In brief, tissue samples were homogenized in 50 mM phosphate buffer containing 14 mM hexadecyltrimethyl ammonium bromide (HTAB; pH 6.0). After two freeze-thaw cycles, samples were centrifuged. After adding the supernatant to an O-dianisidine buffer (50 mM phosphate buffer, 0.52 mM O-dianisidine and 0.15 mM H_2_O_2_), changes in absorbance were measured at 460 nm. Results were expressed as units of MPO per mg tissue, where 1 unit of MPO was defined as the amount of enzyme degrading 1 µmol H_2_O_2_ per minute at 25°C.

### H&E and peroxidase staining

Frozen fundic sections (6 µm) were stained with hematoxylin and eosin. The number of polymorphonuclear (PMN) cells was determined by counting 10 randomly chosen representative high-power fields (40x). The number of peroxidase-positive cells was quantified in sections stained with Hanker Yates reagent (Sigma-Aldrich, St. Louis, MO, USA).

### Statistical analysis

Results are presented as means ± SEM. Data were analyzed using Statistica 11 (StatSoft Inc., Tulsa, OK, USA). Data obtained at different time points in the same mice were analyzed using repeated measures ANOVA. For the EFS-induced responses, post hoc Bonferroni testing was used to detect overall differences between feeding conditions followed by planned comparison testing. Differences between the COEC-fed ALFF and RF groups were detected with an unpaired Student's t-test. All other data were analyzed using a one- or two-way ANOVA, followed by post hoc Bonferroni testing. Significance was accepted at the 5% level.

## Results

### 1. Role of ghrelin in the physiological adaptations induced by RF

#### RF increases plasma ghrelin levels

**Figure 2:**
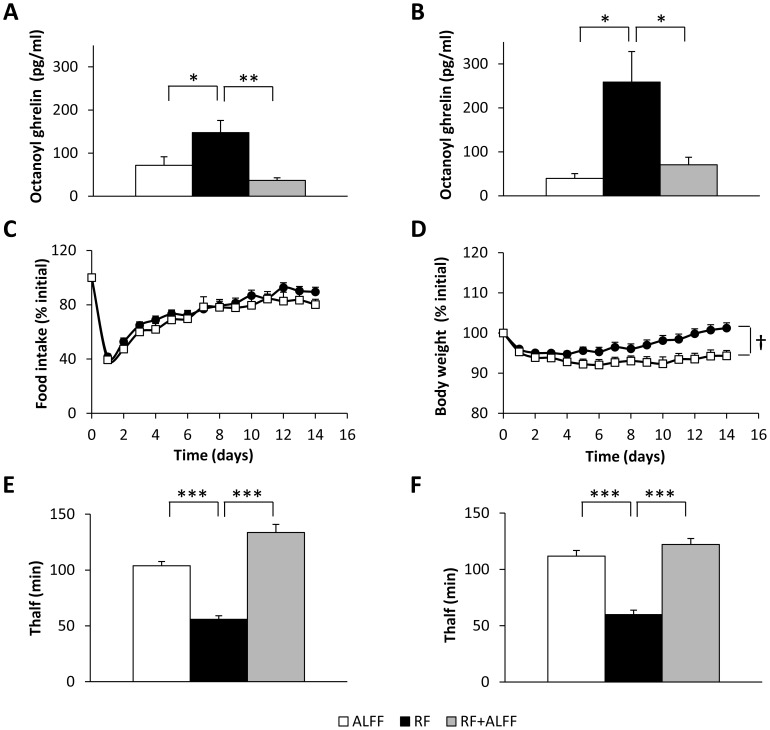
Role of ghrelin in the RF-induced changes in food intake, body weight and gastric emptying. (**A–B**) Plasma octanoyl ghrelin levels in WT (n = 6/group) (**A**) or GHSR-KO mice (n = 2–5/group) (**B**) under ALFF, RF or RF+ALFF conditions. (**C–D**) Food intake and body weight of WT (•)(n = 21) or GHSR-KO (□)(n = 17) mice on RF. (**E–F**) Gastric emptying in WT (n = 12) (**E**) or GHSR-KO mice (n = 10) (**F**) under ALFF, RF or RF+ALFF conditions. *P<0.05,**P<0.01,***P<0.001 vs. RF; **†** *P*<0.05 WT vs. GHSR-KO.

Two weeks of RF increased (*P*<0.05) plasma concentrations of octanoyl (Figure 2*A*) and total ghrelin (RF: 2337±204 vs. ALFF: 1333±243 pg/ml; *P*<0.01) in WT mice. GHSR-KO mice even displayed a 5.3 fold higher rise in octanoyl ghrelin levels (Figure 2*B*). A similar effect was observed on total ghrelin levels (data not shown). In both genotypes, refeeding the mice *ad libitum* for four weeks after two weeks of RF (RF+ALFF) normalized plasma ghrelin levels.

#### RF induces body weight loss

The role of ghrelin signaling in the RF-induced changes in food intake and body weight was studied in WT and GHSR-KO mice. In both genotypes, food intake and body weight dropped to a similar extent at the beginning of the RF schedule (Figure 2 *C–D*). While food intake recovered in both genotypes by the end of the 2-week RF schedule, body weight of GHSR-KO mice remained 9% lower (*P*<0.05) compared to WT mice.

#### RF accelerates gastric emptying

Since ghrelin displays gastroprokinetic properties, gastric emptying rate during RF was assessed. RF accelerated (*P*<0,001) gastric emptying in both WT and GHSR-KO mice to a similar extent, suggesting that this effect is not mediated via the increase in ghrelin levels (Figure 2 *E–F*). Refeeding the mice *ad libitum* (RF+ALFF) normalized gastric emptying in both genotypes.

**Figure 3:**
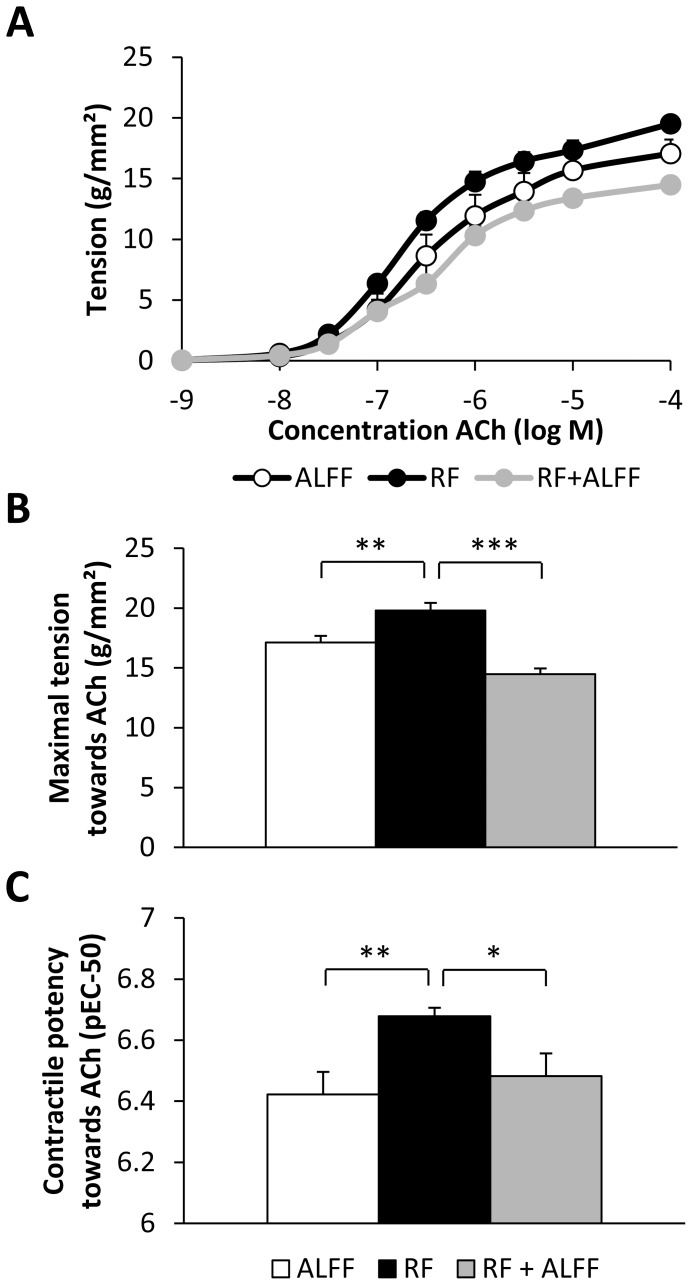
RF with normal chow induces hypercontractility towards ACh in gastric smooth muscle strips. (**A**) Concentration-response curve towards ACh in fundic smooth muscle strips of normal chow-fed WT mice under different feeding conditions. (**B**) Maximal tension and (**C**) contractile potency (pEC-50 value) derived from the concentration-response curves. (ALFF and RF: N = 4 mice, n = 2–3 strips/mouse; RF+ALFF: N = 3 mice, n = 1–3 strips/mouse). * *P*<0.05, ** *P*<0.01, *** *P*<0.001 vs. RF.

**Figure 4:**
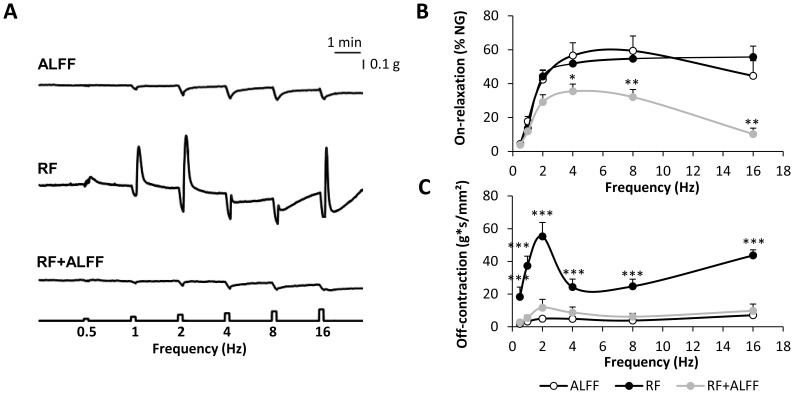
RF with normal chow causes neural hyperexcitability of gastric smooth muscle strips. (**A–C**) Representative tracings of EFS-induced neural responses (**A**) and graphs summarizing changes in on-relaxations (**B**) and post-stimulus off-contractions (**C**) of fundic smooth muscle strips of NC-fed WT mice under ALFF, RF or RF+ALFF conditions (N = 3–4 mice/group, n = 1–3 strips/mouse). *P<0.05, **P<0.01, ***P<0.001 vs. ALFF.

To identify the mechanisms involved in the acceleration of gastric emptying, *in vitro* contractility studies were performed with fundic smooth muscle strips of WT mice. Figure 3A depicts the concentration-response curves towards ACh for the three feeding conditions. RF resulted in a fully reversible increase in maximal tension (*P*<0.01) and contractile potency (pEC_50_) (*P*<0.001) towards ACh (Figure 3 *B–C*). This coincided with a significant increase in the cross-sectional area of the smooth muscle strips (RF: 0.088±0.002 mm^2^ vs. ALFF: 0.073±0.002 mm^2^; *P*<0.01). RF also affected neural responses of the smooth muscle strips elicited by EFS at increasing frequencies of stimulation (Figure 4). There was a significant effect of feeding condition*frequency (*P*<0.001) on the relaxations elicited during the period of stimulation (on-relaxations) and the post-stimulus off-contractions. The EFS-induced on-relaxations did not differ between ALFF and RF mice, but were less pronounced in mice refed *ad libitum* (*P*<0.05 vs. ALFF) (Figure 4*B*). Off-contractions were significantly enhanced (*P*<0.001) in a frequency-dependent manner by RF and normalized again in the RF+ALFF group (Figure 4*C*).

#### RF alters proinflammatory cytokine expression and MPO activity

**Figure 5:**
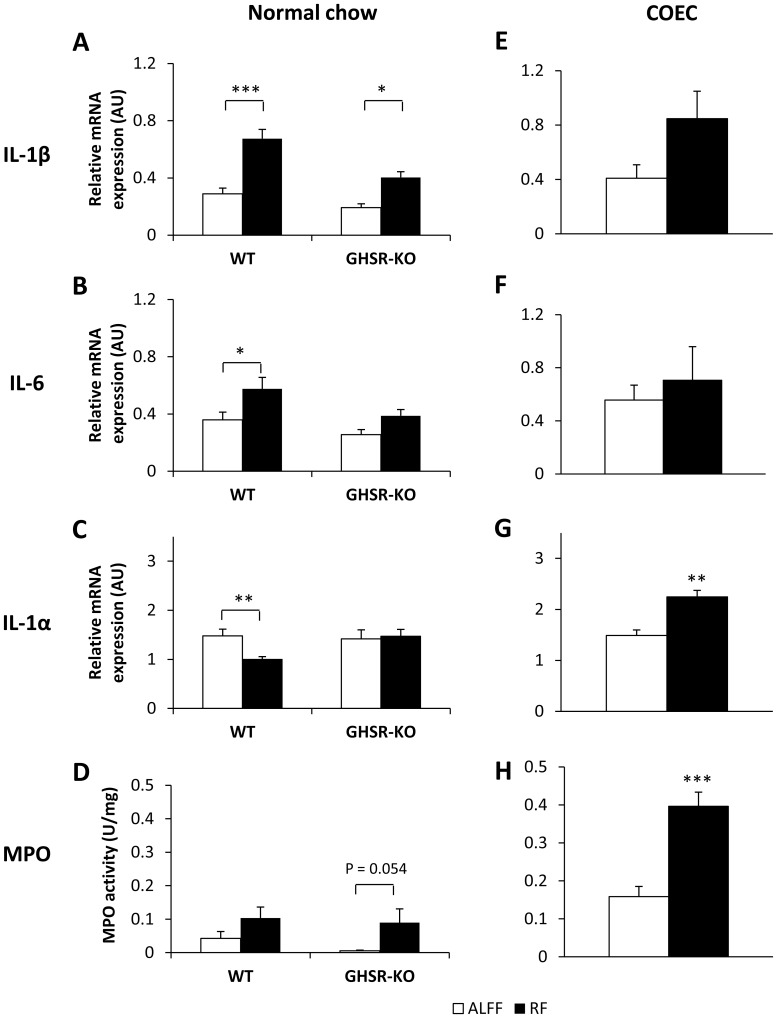
RF with normal chow or COEC alters gastric cytokine mRNA expression and MPO activity levels. Gastric mRNA expression of the proinflammatory cytokines IL-1β (**A**, **E**), IL-6 (**B**, **F**) and IL-1α (**C**, **G**) and MPO activity (**D**, **H**) of normal chow-fed WT (n = 7–16/group) or GHSR-KO (n = 6–9/group) mice (**A–D**) or COEC-fed WT mice (n = 5–6/group) (**E–H**) under different feeding conditions. *P<0.05, **P<0.01, ***P<0.001 vs. ALFF. (AU  =  arbitrary units).

Since ghrelin has been shown to exert anti-inflammatory effects, cytokine expression was quantified in the stomach of WT and GHSR-KO mice. Two-way ANOVA analysis revealed that RF increased IL-1β and IL-6 mRNA expression in the stomach, regardless of the genotype (feeding condition*genotype not significant) (Figure 5 *A–B*). On the contrary, IL-1α mRNA expression was downregulated during RF in WT, but not in GHSR-KO mice (feeding condition*genotype *P*<0.05) (Figure 5*C*). In addition, RF increased MPO activity in both genotypes (feeding condition*genotype not significant) (Figure 5*D*). In both WT and GHSR-KO mice, MPO activity was normalized in the RF+ALFF group (data not shown).

### 2. Role of the core clock gene Bmal1 in the physiological adaptations induced by RF

Since our study in WT and GHSR-KO mice demonstrated that the role of ghrelin in the physiological adaptations to RF was limited to the restoration of body weight loss, our focus shifted to the circadian system itself. As expected, RF mice displayed a 6-fold increase in Per2 and an 8-fold decrease in Bmal1 mRNA expression levels in the stomach (data not shown), indicating a phase-shift in clock gene expression. Subsequently, we aimed to determine the role of the core clock gene Bmal1 in the RF-induced physiological adaptations.

When subjected to RF with normal chow, Bmal1-KO mice failed to adapt to the RF schedule and died within 4 days, due to a persisting decrease in food intake and body weight (data not shown). Therefore, several adjustments had to be made to the RF schedule according to a previous study [17]. In the final scheme (Figure 1*B*), food availability was decreased gradually and during the last 7 days, chow was enriched with canola oil to increase the caloric content of the meal.

However, since a high-fat diet itself is capable of perturbing circadian rhythms at the molecular and behavioral level [21] and stimulating low-grade inflammation [22], we first investigated the impact of the COEC on the physiological adaptations during the standard RF schedule (Figure 1*A*) in WT mice, before imposing the adapted RF schedule onto Bmal1-KO mice.

#### Standard RF schedule with COEC in WT mice

**Figure 6:**
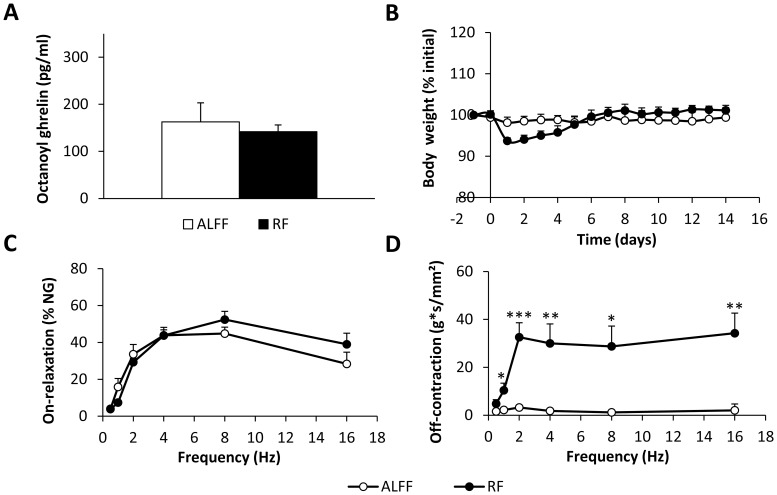
Effect of RF with COEC on plasma octanoyl ghrelin levels, body weight and gastric *in vitro* contractility. (**A**) Plasma octanoyl ghrelin levels, (**B**) changes in body weight and (**C–D**) EFS-induced on-relaxations (**C**) and post-stimulus off-contractions (**D**) of fundic smooth muscle strips of COEC-fed WT mice under ALFF and RF conditions (N = 5–7 mice/group, n = 8–10 strips). *P<0.05, ** *P*<0.01, ***P<0.001 RF vs. ALFF.

In contrast to normal chow, 2 weeks of RF with COEC did not increase plasma octanoyl ghrelin levels (Figure 6*A*). In addition, under fasting conditions (ALFF), plasma octanoyl ghrelin was 127% higher (*P*<0.01) in COEC-fed mice compared to normal chow-fed mice.

COEC-fed mice were able to restore their body weight in less than one week's time, compared to two weeks with normal chow (Figure 6*B*).

RF with COEC tended (*P* = 0.07) to increase the maximal tension (RF: 18.0±0.7 vs. ALFF: 15.1±1.4 g/mm^2^; *P* = 0.07) of the contractile response to ACh in fundic strips. However, in contrast to RF with normal chow, the pEC_50_-value towards ACh was not increased (RF: 6.31±0.04 vs. ALFF: 6.24±0.08). RF also resulted in neuronal hyperexcitability of fundic strips, as reflected by a frequency-dependent increase in the post-stimulus off-contractions (feeding condition*frequency, *P*<0.001) (Figure 6*D*). Inhibitory on-responses were not affected (Figure 6*C*).

In contrast to normal chow, RF with COEC did not significantly (*P* = 0.10) alter IL-1β mRNA expression, but upregulated (*P*<0.01) IL-1α mRNA expression (Figure 5 *E,G*). IL-6 mRNA expression remained unaffected (Figure 5*F*). Moreover, MPO activity was markedly increased (*P*<0.001) after RF with COEC (Figure 5*H*).

#### Adapted RF schedule with COEC in WT and Bmal1-KO mice

**Figure 7:**
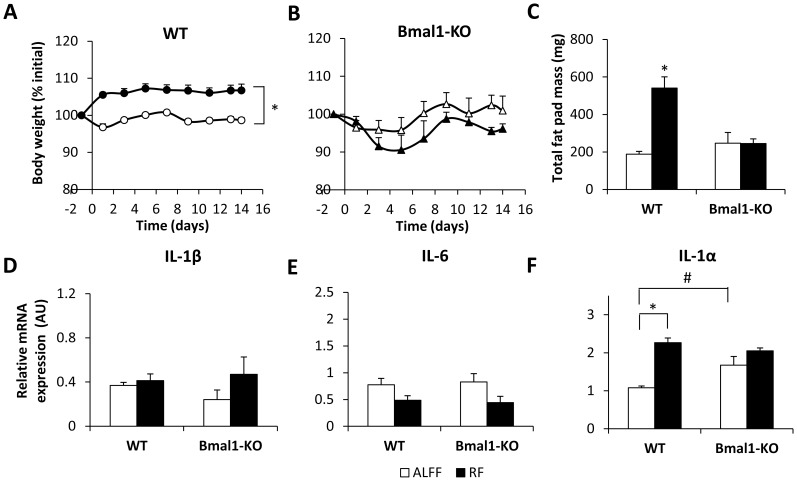
Bmal1 regulates body weight and upregulates gastric IL-1α mRNA expression during RF with COEC. (**A–B**) Changes in body weight of (**A**) COEC-fed WT (n = 11–21/group) or (**B**) Bmal1-KO mice (n = 7–11/group) under ALFF or RF conditions with gradually restricted food availability. (**C**) Total fat pad mass of COEC-fed WT (n = 7–12/group) and Bmal1-KO (n = 6/group) mice under ALFF or RF conditions with gradually restricted food availability. (**D–F**) Gastric mRNA expression of the proinflammatory cytokines IL-1β (**D**), IL-6 (**E**) and IL-1α (**F**) of COEC-fed WT (n = 9–19 mice/group) or Bmal1-KO mice (n = 6–7 mice/group) under ALFF or RF conditions with gradually restricted food availability. *P<0.001 ALFF vs. RF; # *P*<0.01 WT vs. Bmal1-KO.

WT mice on the adapted RF schedule, in which food availability was gradually decreased, did not display a drop in body weight, but immediately outweighed (*P*<0.001) their *ad libitum*-fed counterparts (Figure 7*A*). This coincided with an increase (*P*<0.001) in total fat pad mass, consisting of gonadal and perirenal retroperitoneal fat (Figure 7*C*). In contrast, body weight and total fat pad mass did not differ between ALFF and RF Bmal1-KO mice (Figure 7 *B–C*).

**Figure 8:**
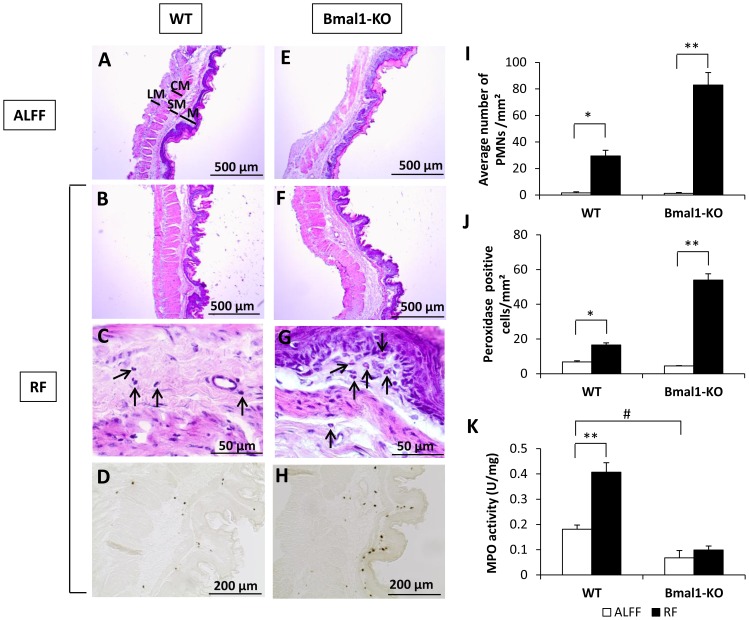
Bmal1 promotes infiltration of neutrophils in the stomach during RF with COEC. Representative H&E stained fundic sections of COEC-fed WT or Bmal1-KO mice under ALFF (**A,E**) or RF (**B,F**) conditions with gradually restricted food availability. (**C,G**) Higher magnifications of RF sections with arrows pointing towards PMNs. Representative peroxidase-stained fundic sections of WT (**D**) and Bmal1-KO (**H**) mice under RF conditions. Average number of PMNs per mm^2^ (**I**) and peroxidase positive cells per mm^2^ (**J**) in the fundus of WT (n = 4–5/group) or Bmal1-KO mice (n = 4/group) under ALFF or RF conditions. (**K**) MPO activity levels of WT (n = 9–20/group) or Bmal1-KO mice (n = 6–7/group) under ALFF or RF conditions. *P<0.01, **P<0.001 ALFF vs. RF; # *P*<0.05 WT vs. Bmal1-KO. (LM  =  longitudinal muscle layer, CM  =  circular muscle layer, SM  =  submucosa, M =  mucosa).

RF led to thickening of the fundic wall, which was more pronounced in the longitudinal muscle layer (Bmal1-KO vs. WT: 100% vs. 30% increase), submucosa (Bmal1-KO vs. WT: 66% vs. 21% increase) and mucosa (Bmal1-KO vs. WT: 49% vs. 23% increase) of Bmal1-KO mice compared to WT mice (Figure 8). The RF-induced increase in the number of inflammatory PMN cells (neutrophils and eosinophils) was more pronounced (genotype*feeding condition *P*<0.001) in Bmal1-KO compared to WT mice (Figure 8*I*). Under ALFF conditions, few peroxidase positive cells (eosinophils, neutrophils and monocytes) were present (Figure 8*J*). The increasing effect of RF on the number of peroxidase positive cells was more pronounced (genotype*feeding condition *P*<0.001) in Bmal1-KO mice. In addition, MPO activity, determined by infiltrating neutrophils and monocytes, was lower (*P*<0.05) in Bmal1-KO mice during ALFF conditions (Figure 8*K*). RF significantly increased MPO activity in WT, but not in Bmal1-KO mice (genotype*feeding condition *P*<0.01). IL-1β and IL-6 mRNA expression did not differ between WT and Bmal1-KO mice during ALFF or RF conditions (Figure 7 *D–E*). In contrast, IL-1α mRNA expression was significantly (*P*<0.01) higher in the stomach of ALFF Bmal1-KO mice (Figure 7*F*). RF significantly upregulated IL-1α mRNA expression in WT, but not in Bmal1-KO mice (genotype*feeding condition *P*<0.01). In summary, these results indicate that Bmal1 promotes infiltration of neutrophils and mRNA expression of IL-1α in the stomach during RF.

**Figure 9:**
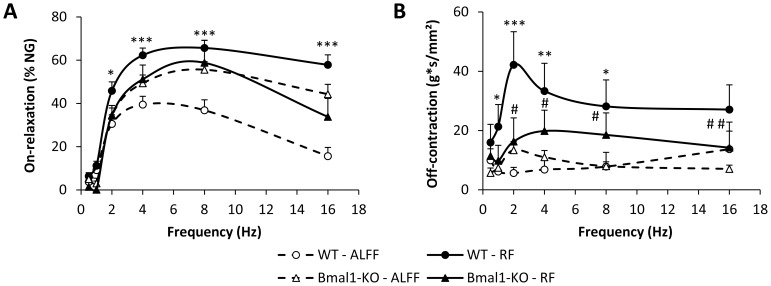
Bmal1 is involved in the contractility changes induced by RF with COEC. (**A**) On-relaxations and (**B**) off-contractions of COEC-fed WT or Bmal1-KO mice under ALFF and RF conditions with gradually restricted food availability. (ALFF: N = 8–14 mice/genotype, n = 16–17 strips; RF: N = 4–8 mice/genotype, n = 6–12 strips). *P<0.05, **P<0.01, ***P<0.001 WT vs. ALFF; # *P*<0.05, ## *P*<0.001 Bmal1-KO vs. ALFF.

In both genotypes, RF increased (*P*<0.05) the maximal tension (WT: RF 17.1±0.9 vs. ALFF 14.0±1.0 g/mm^2^, *P*<0.05; Bmal1-KO: RF 17.0±0.5 vs. ALFF 14.4±0.2 g/mm^2^, *P*<0.05) towards ACh to a similar extent. The RF-induced increase in contractile potency (pEC_50_) towards ACh in WT mice (RF: 6.42±0.05 vs. ALFF: 6.22±0.05; *P*<0.05) was abolished in Bmal1-KO mice (RF: 6.20±0.06 vs. ALFF: 6.12±0.04). Under ALFF conditions, EFS-induced on-relaxations were more pronounced in Bmal1-KO than in WT mice (genotype, *P*<0.05) (Figure 9*A*). In WT, but not in Bmal1-KO mice, RF enhanced the on-relaxations (feeding condition*frequency, *P*<0.001). Similarly, neural off-contractions were increased (feeding condition*frequency, *P*<0.001) after RF in WT mice (Figure 9*B*). This increase was less pronounced in Bmal1-KO mice (feeding condition*frequency *P*<0.01).

## Discussion

**Figure 10:**
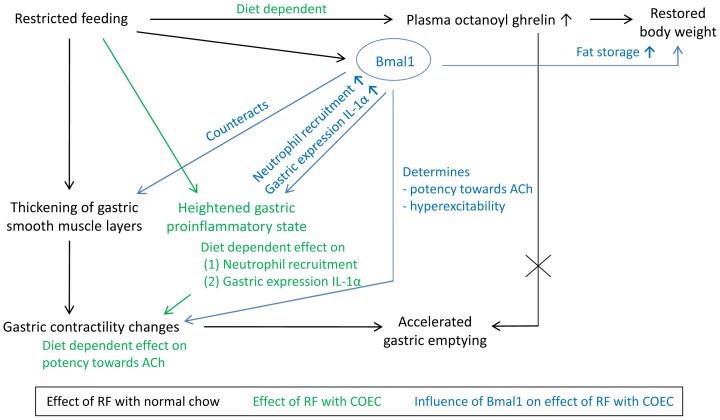
Schematic overview of the role of ghrelin and the clock gene Bmal1 in the physiological adaptations induced by RF with different diets, half an hour prior to food availability.

Circadian clocks are widely accepted to act as the timekeeping system of nearly every living organism, ensuring that physiological processes are carried out at the right time of day or night. However, the concept that the gut is home to one of the food-entrainable oscillators that predicts the availability of food, especially when food becomes restricted, is rather new in the field. Our aim was to study the role of ghrelin and the core clock gene Bmal1 in the physiological adaptations to an altered circadian rhythm of feeding, induced by RF. We show that the food-anticipatory increase in ghrelin during RF depends on the type of nutrients ingested. Furthermore, we demonstrate that during RF, ghrelin and Bmal1 are involved in the restoration of body weight. We also provide the first evidence that RF alters cytokine mRNA expression levels in the stomach prior to food availability. A high-fat diet modulates this expression profile and results in a heightened proinflammatory state in the stomach right before food becomes available. Moreover, the type of inflammatory cells infiltrating the stomach is modulated by Bmal1, which specifically promoted the infiltration of neutrophils and upregulated IL-1α expression. RF also results in a thickening of the smooth muscle layers, which is counteracted by Bmal1. Our findings suggest that this thickening, whether or not in combination with the proinflammatory state, but not ghrelin signaling, leads to hyperexcitability of gastric smooth muscle, thereby promoting gastric emptying during RF. These findings are schematically represented in Figure 10.

We acknowledge that data collection and analysis of only a single time point is a limitation of this study. However, due to several practical reasons, obtaining data at additional time points was not feasible. Nevertheless, our data underpin a clear link between the circadian, metabolic, gastrointestinal and immune system and emphasize that not only “what” but also “when” you eat may determine whether you are prone to develop inflammation, gastrointestinal symptoms or obesity.

We showed that changes in plasma ghrelin levels during fasting (ALFF) were nutrient-dependent. Although it is well established that ghrelin levels are decreased during obesity, fasting levels in mice with *ad libitum* access to COEC for two weeks were twice as high as in mice fed normal chow. Similar observations were made in mice fed a high-fat diet for two weeks and in humans that were subjected to overfeeding during one week [23], [24]. Since a high-fat diet can phase-shift and alter the amplitude of clock genes and clock-controlled humoral and central metabolic systems [21], [25], we propose that the higher fasting plasma ghrelin levels originate from the clock altering capacities of the COEC.

Similar to previous studies [11], [12], we showed that RF with normal chow increased plasma octanoyl ghrelin levels of mice 30 minutes before food anticipation, an effect that can be reversed by refeeding *ad libitum*. Bodosi *et al*. previously demonstrated that this increase was the consequence of both a phase-shift and an increase in the amplitude of the circadian rhythm of ghrelin levels [12]. However, since ghrelin signaling may not be necessary for food-anticipatory activity, ghrelin might either be redundant with other (signals entraining) FEOs or act downstream of or in parallel with other FEOs that drive this activity [13]. Use of the COEC abolished the RF-induced increase in octanoyl ghrelin levels. We speculate that the high-fat feeding resulted in a lack of phase-shift or difference in amplitude between the diurnal rhythms of the plasma ghrelin levels of the ALFF and RF groups. However, this remains to be investigated further.

Our studies in GHSR-KO mice showed that ghrelin signaling was important to restore body weight loss during RF with normal chow. Ghrelin does not only affect body weight through its orexigenic effect, but also by preventing the utilization of fat [26]. Martínez-Merlos *et al*. demonstrated that RF induced epididymal and perirenal retroperitoneal fat loss in rats [27]. Since we did not observe any changes in food intake between WT and GHSR-KO mice, we speculate that the increased ghrelin levels during RF were not able to stimulate adipogenesis in GHSR-KO due to the absence of a functional ghrelin receptor.

Mice subjected to RF with COEC during 4 hours for 2 weeks restored their body weight much faster than normal chow-fed mice. When the availability of the COEC during RF was gradually decreased, mice were protected from entering a negative energy balance, allowing them to quickly outweigh their *ad libitum* fed counterparts. Arble *et al*. showed that mice fed a high-fat diet only during the light-phase, similar to our study, gained significantly more weight than mice fed solely during the dark-phase [28]. Similarly, Mistlberger *et al.* demonstrated that weight gain in a genetic model of obesity was reduced by preventing food intake in the light-phase, without reducing total daily caloric intake [29]. These results again stress the importance of the timing of food intake. Indeed, people that consume most of their food during their normal sleep period – such as shift workers, breakfast skippers and individuals suffering from night eating syndrome – suffer from obesity and related metabolic diseases more frequently [30], [31], [32]. However, in contrast to the findings in short-term studies like ours, weight gain resulting from high-fat feeding in mice can be prevented by restricting food availability to the dark-phase or even the light-phase during a long-term period of 18 weeks [33], [34].

Similar to timing of food intake, deleting circadian clock genes in mice can drastically affect body weight and composition [16], [35], [36], [37]. Here, we show that the increase in body weight during RF with COEC was regulated by Bmal1, since Bmal1-KO mice were protected from the increase in body weight and fat pad mass. This again highlights the crucial role of Bmal1 in the control of adipogenesis and lipid metabolism [38], [39].

Ghrelin is an important gastroprokinetic agent and accelerates gastric emptying in humans and rodents [7]. Ariga *et al.* hypothesized that the RF-induced increase in ghrelin might be responsible for the acceleration in gastric emptying in rats [40]. We showed that RF reversibly accelerated gastric emptying in both WT and GHSR-KO mice, indicating that this acceleration is independent from ghrelin signaling. Our *in vitro* contractility studies in fundic smooth muscle strips demonstrated that RF with normal chow resulted in fully reversible neural hyperexcitability and hypercontractility to ACh. Thus, RF promotes numerous metabolic and physiological adaptations to optimize biochemical handling of a sudden increase in nutrients. In addition, our results show that Bmal1 is involved in the RF-induced increase in contractile potency towards ACh and the neural hyperexcitability.

Circadian regulation has been described for levels of circulating proinflammatory cytokines, different populations of leukocytes, but also for NK cell function and phagocytic activity by macrophages and neutrophils [41]. The current study indicates for the first time that RF probably shifts the rhythm of cytokine mRNA expression and infiltrating immune cells in the stomach, as evidenced by the altered gastric proinflammatory cytokine expression profiles and MPO activity levels. This is strengthened by the study of Sherman *et al.* which showed that long-term (4 months) RF altered mRNA expression levels of proinflammatory cytokines in the liver, jejunum and white adipose tissue [42].

In addition, we show that it is likely that RF with a high-fat diet further shifts this rhythm, leading to increased MPO activity and IL-1α mRNA expression at the expense of IL-1β, which was upregulated by RF with normal chow. Our histological studies confirm that RF with a high-fat diet induces a heightened proinflammatory state in the stomach half an hour before food availability, characterized by infiltrating immune cells. This adds to the finding of Luna-Moreno *et al.* that RF was capable of inducing local immune responses in the liver [43].

Furthermore, Bmal1 seems to upregulate IL-1α expression in the stomach during RF with COEC. In addition, we showed that the type of inflammatory infiltrate is altered in the absence of Bmal1. Indeed, the results of the PMN cell and peroxidase positive cell countings in combination with the MPO activity assay showed that Bmal1 is involved in the recruitment of neutrophils, but not of eosinophils, to the stomach during RF. Since the circadian and immune systems are closely intertwined [41], it is not surprising that genetic circadian disruption can modulate innate immunity. Indeed, Bmal1-KO mice suffer from progressive corneal inflammation [44]. Also, myeloid cell-specific Bmal1 deletion induces expression of monocyte-attracting chemokines and disrupts rhythmic trafficking of proinflammatory Ly6C^hi^ monocytes, leading to predisposition to inflammation-associated pathologies [45]. In addition, recent evidence has emerged for a role of clock genes in natural killer cell and neutrophil function after activation of innate immune responses [46], [47].

Our histological studies indicate a thickening of the fundic smooth muscle layers during RF which may contribute to the hypercontractility of the stomach and the accelerated gastric emptying, in order to adequately process the large amounts of food ingested during the period of food availability. Bmal1 seems to play a pivotal role in this process, since the thickening of the fundic longitudinal muscle layer during RF was more pronounced in Bmal1-KO mice than in WT mice. Nevertheless, we cannot exclude that the expressed cytokines are also contributing to this hypercontractility and neural hyperexcitability. Several studies provide a causal relationship between the presence of cytokines in the gastrointestinal tract and alterations of intestinal transit and smooth muscle contractility. The proinflammatory cytokine IL-1β has been shown to enhance fundic smooth muscle contractility and to induce hyperexcitability of neurons of the myenteric plexus [48], [49]. On the contrary, TNF-α inhibited contraction of ileal smooth muscle tissue of mice [50]. Similarly, IFNγ decreased carbachol-induced longitudinal smooth muscle contraction [51]. To our knowledge, there have been no studies reporting the influence of IL-1α on smooth muscle contractility. The upregulation of this cytokine might offer an explanation for the hypercontractility observed during RF with COEC.

In conclusion, our findings provide a clear link between the circadian, gastrointestinal, immune and metabolic system. This interconnection may provide a rationale why disruption of biological rhythms in shift workers or frequent time zone travelers not only results in obesity and its associated comorbidities, but also in immune-related gastrointestinal pathologies such as gastric ulcers and irritable bowel syndrome, which is often associated with low-grade inflammation [52].
